# Comparison of the Sequence-Dependent Fluorescence of the Cyanine Dyes Cy3, Cy5, DyLight DY547 and DyLight DY647 on Single-Stranded DNA

**DOI:** 10.1371/journal.pone.0085605

**Published:** 2014-01-15

**Authors:** Nicole Kretschy, Mark M. Somoza

**Affiliations:** Institute of Inorganic Chemistry, University of Vienna, Vienna, Austria; University of North Carolina at Charlotte, United States of America

## Abstract

Cyanine dyes are commonly used for fluorescent labeling of DNA and RNA oligonucleotides in applications including qPCR, sequencing, fluorescence *in situ* hybridization, Förster resonance energy transfer, and labeling for microarray hybridization. Previous research has shown that the fluorescence efficiency of Cy3 and Cy5, covalently attached to the 5′ end of single-stranded DNA, is strongly sequence dependent. Here, we show that DY547 and DY647, two alternative cyanine dyes that are becoming widely used for nucleic acid labeling, have a similar pattern of sequence-dependence, with adjacent purines resulting in higher intensity and adjacent cytosines resulting in lower intensity. Investigated over the range of all 1024 possible DNA 5mers, the intensities of Cy3 and Cy5 drop by ∼50% and ∼65% with respect to their maxima, respectively, whereas the intensities of DY547 and DY647 fall by ∼45% and ∼40%, respectively. The reduced magnitude of change of the fluorescence intensity of the DyLight dyes, particularly of DY647 in comparison with Cy5, suggests that these dyes are less likely to introduce sequence-dependent bias into experiments based on fluorescent labeling of nucleic acids.

## Introduction

Fluorescent readout from labeled nucleic acids on solid surfaces or in solution is a common element in a broad range of biotechnological and biophysical methodologies. In most cases, such as in microarray experiments, sequencing-by-synthesis, qPCR, and fluorescence *in situ* hybridization (FISH), the objective is to quantity the abundance of the labeled molecule. In the case of Förster resonance energy transfer (FRET), the magnitude of the transfer of fluorescence energy can be used to determine the distance and/or relative angular orientations between the donor and acceptor. In all of these cases, changes in the fluorescence efficiency of the dye, due to sequence-specific interactions with the labeled strand of DNA or RNA may introduce biases into measurements, either because the measured fluorescence intensity is not proportional to the number of labeled molecules or, in the case of FRET, because the nucleobases between the donor and acceptor are modulating the intensity via an alternative physical process. Previous experiments have shown that the fluorescence of the cyanine dyes Cy3 and Cy5, which are commonly used in nucleic acid labeling applications, are very sensitive to their nucleobase environment, both to nucleobases in solution [1], and covalently bound to the 5′ termini of both single- [2], [3] and double-stranded DNA [4].

The cyanine dyes are highly fluorescent molecules that can be modified to cover a wide spectral range, allowing for multiplexing in high-throughput applications. Unlike other classes of dyes, such as the fluorescein and rhodamine derivatives, cyanine dyes are not quenched by photoinduced charge-transfer interactions with nucleobases, but they are vulnerable to loss of fluorescence due to excited state *cis*-*trans* isomerization about the linkage between the two indole rings [5]. Cy3 is known to bind to nucleobase monophosphates in solution, and both Cy3 and Cy5 have been shown to stack on the end of double-stranded DNA, like a terminal base pair [6], [7]. This affinity appears to be driven by π-stacking interactions with nucleobases, which also restricts the rotational isomerization of the dyes and increases their fluorescence. The mechanism responsible for sequence-specific fluorescence of oligonucleotides labeled with cyanine dyes is not known, but more rigid base stacks may enhance the ability of the terminal nucleobase to hinder dye isomerization. The rigidity of the base stack is largely determined by its purine content [8], [9] because purines have a larger stacking area and higher free energy for stacking [10], [11], [12].

Our previous results for sequence-dependent fluorescence of Cy3 and Cy5 covalently bound to the 5′ end of ssDNA demonstrated that a high purine content results in high intensity analogously to how high GC content results in high melting temperature for complementary sequences; the GA or CT content function almost as random variables, leading to probability distributions that are close to normal distributions [2]. Superimposed on this pattern is, in the brightest sequences, an overrepresentation of dG at the 5′end and an overrepresentation of dA in subsequent positions, and an overrepresentation of dC at the 5′end of the darkest sequences. Experiments with the same sequences, but with a 5′ biotin phosphoramidite and subsequent labeling with Cy3- or Cy5- conjugated streptavidin, resulted in a much stronger sequence-dependent fluorescence [2]. One possible explanation for the differences between the results with direct labeling with dye phosphoramidites and indirect labeling via dye-streptavidin-biotin conjugates is that the dye-DNA interactions are highly sensitive to apparently minor changes to the dye structure or tethering mechanism.

**Figure 1:**
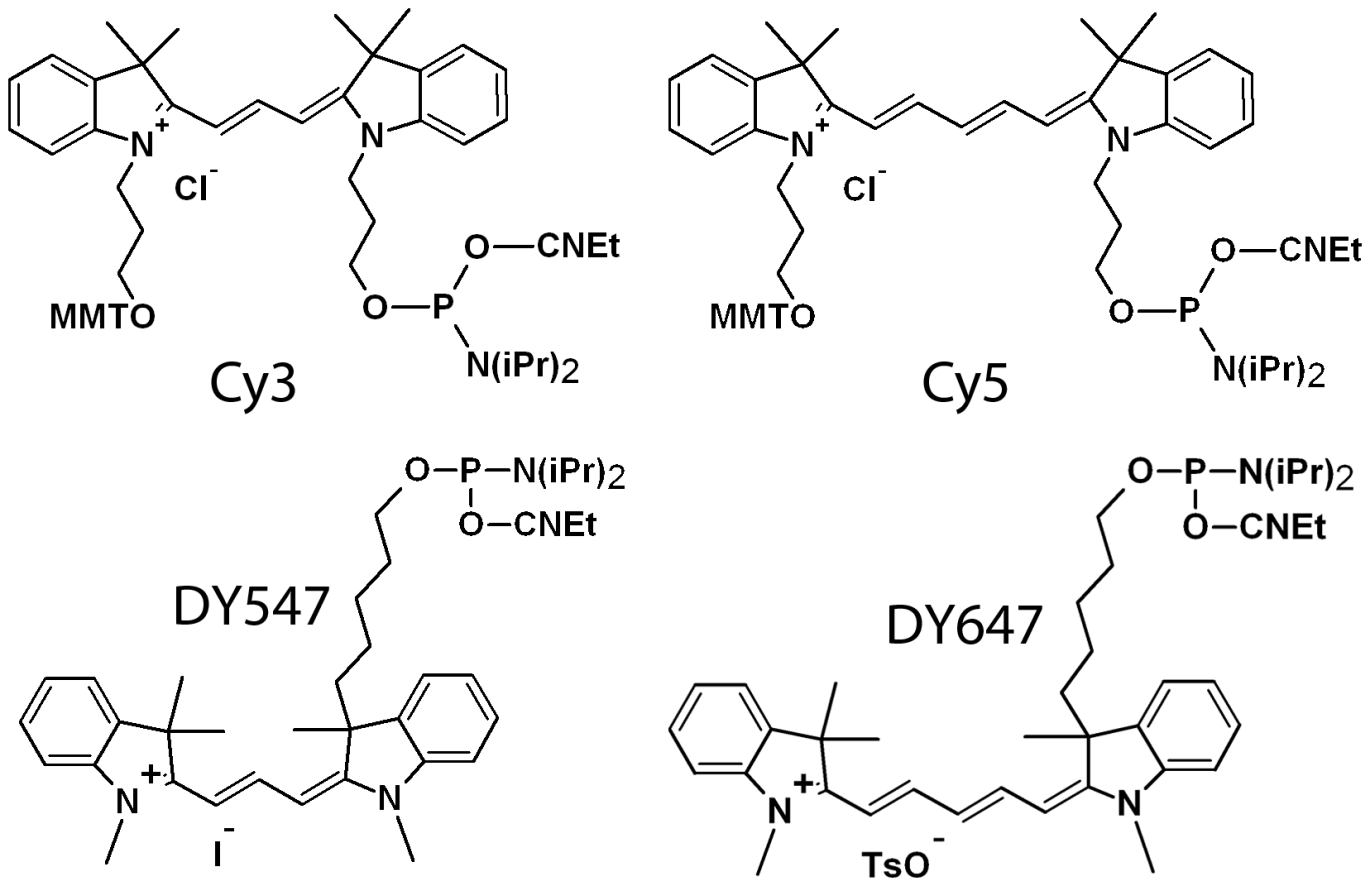
Molecular structures of the cyanine dye phosphoramidites used in this study: Cy3, Cy5, DY547 and DY647. Monomethoxytrityl (MMT) groups are present on the Cy-dyes; the 2-cyanoethyl group (CNEt) is the standard phosphate protecting group in oligonucleotide synthesis, and the diisopropyl group (N(iPr)_2_) is displaced during the coupling reaction by the 5′-hydroxyl group to form a phosphate linkage between the terminal nucleoside and the dye.

The experiments presented here were motivated by an interest to establish the parameters affecting the sequence-dependent fluorescence of cyanine dyes. The DyLight cyanine dyes DY547 and DY647 are structurally similar to Cy3 and Cy5, but differ in how they are tethered to the DNA (Figure 1). In addition, the Cy3 and Cy5 phosphoramidites also include the monomethoxytrityl (MMT) group to allow either 3′ labeling, or reverse-phase HPLC purification. The MMT group may affect how the dyes interact with DNA. We were also interested in evaluating whether DY547 and DY647 can be used as direct replacements for Cy3 and Cy5 in sensitive terminal-labeling experiments. Some labeling applications, such as those based 5′-labeled random primers, or amino allyl-dUTP or dye-dNTPs labels randomly incorporated during reverse transcription, should be mostly insensitive to sequence-dependent fluorescence, due to the quasi-random nature of the labeling, but in methods based on labeling of specific sequences, changes in the sequence dependency would affect the results. Beyond improving the accuracy of experiments based on fluorescence labeling of nucleic acids, an understanding of the sequence-dependency of dyes may lead to insights into sequence-specific biophysical properties of nucleic acids, such as DNA rigidity, which affects DNA-protein interactions [13], [14], [15].

## Methods

The *in situ* synthesis of microarrays, including combinatorial arrays of fluorescently labeled ssDNA has been described in detail previously [2], [16], [17], [18], [19], [20]. Briefly, maskless array synthesis (MAS) [21], was used to produce microarrays with 20 or 21 replicates of each of the 1024 5′-labeled experimental sequences. Dye phosphoramidites (Figure 1) were purchased from Glen Research. In order to produce data unbiased by the different coupling efficiencies of the four DNA phosphoramidites, the following sequence design was used:

### 5′-(dye)-*N*_1_ *N*_2_ *N*_3_ *N*_4_ *N*_5_-T_15_-(ACGT-*N*_1_)-(ACGT-*N*_2_)-(ACGT-*N*_3_)-(ACGT-*N*_4_)-(ACGT-*N*_5_)-T_5_-(surface)

The ***N*** **_i_** represent the 5-mer experimental sequences. These are separated by a 15-thymidine linker from a 15-mer with bases customized as shown, that is, with each of the experimental bases subtracted from five copies of sets of all four DNA bases. This design, in conjunction with acetic anhydride capping following each coupling, ensures that all of the sequences which receive the 5′-dye will have the design sequence, which includes the same number of each base, and hence, each experimental sequence has equal number density on the microarray surface. The 15-thymidine linker was chosen to minimize possible long-distance through-the-stack interactions with the downstream nucleobases used to ensure homogenize sequence number density.

After synthesis, the microarrays were vigorously washed for 2 h with acetonitrile to remove traces of dye phosphoramidite from the glass surface. Protecting groups were removed in 2 h with a 1∶1 (v/v) solution of ethylenediamine in ethanol. The microarrays were then washed twice with distilled water, dried with argon and immediately scanned using GenePix 4100A. Fluorescence intensity values were extracted from the scan images using NimbleScan v2.1. The fluorescence intensity values were calculated as the average of the 20 or 21 replicates of each sequence, which were randomly arranged on each microarray. Error was calculated as the standard error of the mean (SEM). The consensus sequence logos were generated by ranking the 1024 sequences by fluorescence intensity and then dividing the sequences into eight bins spanning equal ranges of intensity. Consensus logos for each of these octiles of fluorescence intensity were generated using Weblogo (weblogo.berkeley.edu) [22].

## Results and Discussion

**Figure 2:**
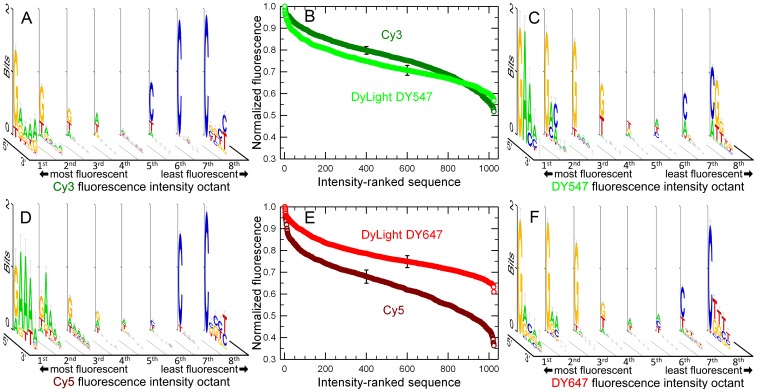
Fluorescence intensity of cyanine-labeled single-stranded DNA. (A) Fluorescence intensity consensus sequence logos of all 1024 ssDNA 5-mers labeled with a 5′ Cy3 phosphoramidite. Each consensus logo corresponds to those sequences spanning one eighth of the intensity range. (B) Fluorescence intensity of Cy3 and DY547 end-labeled 5-mers, ranked from most to least intense. The Cy3 curve drops by about 50% of the maximum intensity, while the DY547 curve drops about 45%. (C) Consensus sequence logos of all 1024 ssDNA 5-mers labeled with a 5′ DY547 phosphoramidite. (D) Consensus sequence logos of all 1024 ssDNA 5-mers labeled with a 5′ Cy5 phosphoramidite. (E) Fluorescence intensity of Cy5 and DY647 end-labeled 5-mers, ranked from most to least intense. The Cy5 curve drops by about 65% of the maximum intensity, while the DY647 curve drops about 40%. (F) Consensus sequence logos of all 1024 ssDNA 5-mers labeled with a 5′ DY647 phosphoramidite. The single error bars (SEM) on each curve are representative. The z-axis height measures the information content at each site in units of bits.

All four of the cyanine dyes studied, Cy3, Cy5, DY547 and DY647, interact very similarly with DNA. For all of the dyes, the consensus sequences resulting in the highest fluorescence begin with a 5′ guanine followed by multiple adenines; the consensus sequences resulting in the lowest fluorescence always start with a 5′ cytosine (Figure 2). While the consensus logos are quite similar for all of the dyes, there are some differences between the Cy-dyes and the DY-dyes. Adenine is almost never found in the least fluorescent Cy-labeled sequences, but does appear in the least fluorescent DY-labeled sequences, particularly in the case of DY547. Cytosine is more common among the brightest DY-labeled sequences compared to the Cy-labeled sequences. Conversely, thymine is often found among the brightest Cy-labeled sequences, but rarely among the brightest DY-labeled sequences.

Although all of the dyes have similar sequence dependence, the magnitude of change of the fluorescence intensity of the DY dyes, over the range of all 1024 sequences, is smaller than that of the Cy dyes, particularly Cy5 (Figure 2B & E). The fluorescence intensity of DY547 falls by ∼45% from the brightest (GAAAA) to the least bright sequence (CGTGT). By comparison, the intensity of Cy3 falls slightly more, ∼50% over the same range. In the case of Cy3, the brightest of all 1024 sequences is also GAAAA, but the darkest, CGGTT, is similar but not identical. The dye DY647 has the smallest range of fluorescent intensity, which drops by ∼40% over the range of all 5-mers. The brightest of the DY647 sequences is GGGGT, highlighting that the consensus sequence for the brightest DY647-labeled DNA oligomers is different from those of the other three dyes (all **GAAAA**); specifically, the 5′-guanine remains important, but adenines are not dominant in subsequent positions. The darkest Cy5- and DY647-labeled sequence are CGGTC and CTTTT, respectively. In the case of DY647, the darkest sequence is an exact match to the consensus sequence for the lowest octant of fluorescence. The fluorescence intensity of all 1024 5-mers for all four dyes is provided as Data S1 in spreadsheet format as Supporting Information. The individual logos used to make Figure 2 are shown in Figure S1.

We have previously hypothesized that sequence-specific fluorescence results from stacking interactions that modulate the rate of rotational isomerization. The current data is consistent with that hypothesis. Guanine has the largest calculated stacking area, based on B-form stacking geometry: dG (139 Å^2^)>dA (128 Å^2^)>dC (102 Å^2^)>dT (95 Å^2^) [11], and cyanine dyes have the greatest fluorescence in solution along with dG homopolymers [23] and in solution with guanosine monophosphates [1], relative to the other three nucleobases. This suggests that a 5′ guanosine is important for fluorescence because this base preferentially stacks with cyanine dyes and restricts fluorescence quenching due to rotational isomerization. Based on the stacking area calculations, a 5′ thymine would be predicted to result in the lowest fluorescence, but both the homopolymer and nucleoside monophosphate data indicate that cytosine results in the lowest fluorescence of cyanine dyes, in agreement with all the data presented here. The more distal nucleobases may stabilize or destabilize the interaction of the 5′ nucleobase with the dye. It is known that purine stacks in single-stranded DNA are more rigid that pyrimidine stacks and that mixed purine-pyrimidine stacks have intermediate rigidity [8], [9]. Distinctions between purines or between pyrimidines are more ambiguous. The stacking energy ΔΔG°, based on ssDNA to dsDNA equilibrium experiments, follows the order dA>>dG>dT≈dC (2.0, 1.3, 1.1 and 1.0 kcal/mol, all ±0.2) [11], which is consistent with the dominance of adenine in distal positions of the consensus logos for the brightest sequences. However, experiments based on 3′ dangling bases have indicated that dA and dG stabilize the stack approximately equally [10], [12]. Although the hypothesis is that stacking interactions are responsible for the observed sequence-dependent fluorescence, deviations from the expected trend suggest that other mechanism also influence dye intensity. For example, dT and dC are occasionally present in the brightest Cy-dye and DY-dye sequences, respectively, and although pyrimidines dominate the darkest sequences, dG is also relatively abundant in distal positions, and DY547 even has a significant representation of dA at the 5′ position.

Another of the objectives of the project presented here was to evaluate if the DY-dyes can be used to replace Cy-dyes in experiments that may be sensitive to sequence-dependent fluorescence, such as using fluorescence intensity to quantify the relative abundance of specific nucleic acid sequences. The absorption and emission spectra of Cy3 and DY547, and Cy5 and DY647 are essentially identical, and the DY-dyes have a slightly higher quantum yield [24], but large differences in the pattern of sequence-dependent fluorescence could result in shifts of the relative intensities when substituting dyes. The results presented here indicate that intensities of some labeled sequences would shift, for example, the sequences Cy3-TATAA and Cy5-TATAA are among the brightest, 2^nd^ and 13^th^ brightest, respectively, while DY547-TATAA and DY647-TATAA are significantly darker, with a rank of 81^st^ and 242^nd^, respectively. Nevertheless, since the overall consensus sequence patterns are similar, the relative intensities of most sequences would only change modestly. A significant motivation for using the DY-dyes instead of the Cy-dyes is that the intensity difference between the brightest and darkest sequences are smaller, particularly in comparison with Cy5, so that the probability that a randomly chosen sequence will result in poor fluorescence is lower. To some extent, even applications based on random labeling, for example, genomic DNA or RNA labeling using 5′-labeled random nonamers [25], are subject to sequence-dependent fluorescence biases due to the variable nucleobase content of genes [26]. Quantitative PCR experiments based on fluorescent reporter oligonucleotides (Molecular beacon or TaqMan probes) are significantly more vulnerable to sequence-dependent fluorescence since a single reporter sequence is chosen for each reaction. Standard curves can at least partially compensate for such biases, but poorly fluorescent reporter probes will inevitably degrade data quality. High-throughput DNA sequencing-by-synthesis is likely to be particularly vulnerable to sequence-dependent fluorescence because all short nucleobase sequences will be repeatedly encountered, and detection failures (deletion errors) from sequences highly unfavorable to fluorescence would be systematic and therefore not detectable with re-sequencing. Furthermore, the optical systems of sequencers need to balance dynamic range of detection with throughput, making them vulnerable to dyes with significant variations in fluorescence [27].

In conclusion, combinatorial microarrays of labeled DNA can effectively determine patterns of sequence-dependent fluorescence. Applying this method to commonly used cyanine dyes indicates that DY547 and DY647 are less likely to result in sequence-dependent labeling artifacts in comparison to Cy3 and Cy5. While many properties of dyes, such as quantum yield, photostability and sensitivity to a variety of environmental factors, can affect signal intensity, sequence-dependent fluorescence may be more likely to introduce systematic biases into experimental results.

## Supporting Information

Figure S1 **Fluorescence intensity consensus logos from** **Figure 2** **.** Individual consensus logos for Cy3, DY547, Cy5 and DY647. From left to right, the logos represent each of the eight bins in order of decreasing fluorescence intensity.
(TIF)

Data S1 **Fluorescence intensity data (sequence, normalized intensity, SEM) for all labeling methods in spreadsheet format.**
(XLS)
