# Supplementary figures and images for: Comparison of the Sequence-Dependent Fluorescence of the Cyanine Dyes Cy3, Cy5, DyLight DY547 and DyLight DY647 on Single-Stranded DNA
Source: PLoS One. 2014 Jan 15;9(1):e85605. doi: 10.1371/journal.pone.0085605 (PMC3893224; doi:10.1371/journal.pone.0085605)

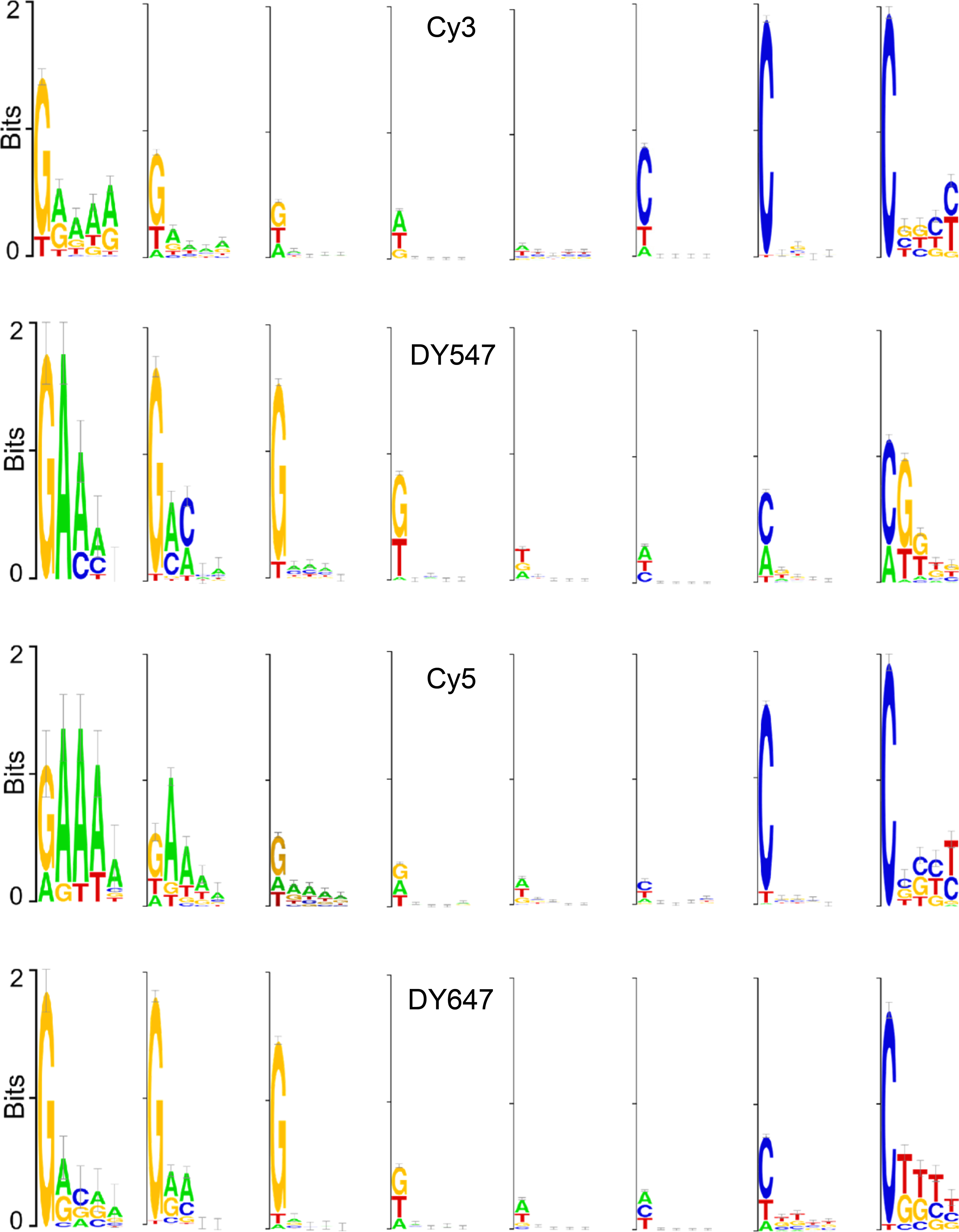

Supplement: Figure S1 — Fluorescence intensity consensus logos from Figure 2 . Individual consensus logos for Cy3, DY547, Cy5 and DY647. From left to right, the logos represent each of the eight bins in order of decreasing fluorescence intensity. (TIF) [file pone.0085605.s001.tif]
